# JMP enters a new era

**DOI:** 10.4103/0971-6203.25663

**Published:** 2006

**Authors:** A. S. Pradhan

**Affiliations:** Resident Editor, Journal of Medical Physics, C/o. RP & AD, CT & CRS, Anushaktinagar, Mumbai - 400 094, India. E-mail: editor@jmp.org.in

A quarterly publication of the Association of Medical Physicists of India (AMPI), which started in the year of 1976 in the form of AMPI Medical Physics Bulletin under the dynamic Editorship of Dr. U Madhvanth, took a quantum leap forward to become *Journal of Medical Physics* (JMP) in the year 1996. During the past two decades, the technology has changed dramatically not only in the science of medical physics but in the technique of journal production, promotion and presentation of scientific contents. The days of journal printers working on the camera-ready copy are long over. Now everything is electronic and therefore, the up-gradation of the journal became overdue. Having accepted the responsibility of Resident Editorship of JMP with effect from January 2006, an effort was made to publish JMP online. For this, the publication of JMP is entrusted to a publisher – Medknow Publications Pvt. Ltd.

I am delighted to see the publication of JMP with a new look that incorporated the elements of original design and a new layout of printing format. The most attractive development for the journal is that the full text is now published online, in addition to the printed hard copies. Readers are encouraged to visit the website of the journal. The new development is the use of web-based online submission and peer review system for the manuscript submission. The authors can submit the papers easily from anywhere in the world and then track the progress of the manuscripts within the system. It is needless to point out that publication online will enable the journal to reach a much wider readership than has been possible with the print alone. The online journal can be networked within a subscribing institution, permitting the readers to search and browse on their desk.

For the success of the journal, it is necessary to gather together a highly reputable Editorial Board, covering all topics of the scope of the journal. The members of the Editorial Board, who are from different parts of the world, are well known for their expertise in their respective fields of Medical Physics. Many of these continue from the former Board of Editors with a few new additions.

A major change in the policy of refereeing is that these members of the Editorial Board undertake the refereeing of most of the manuscripts and hunting for a referee outside the Board, with little obligation to JMP, is minimized. The commitment of the members of the Board for refereeing is bound to enhance the standard and the speed of publication. All papers, whether invited or contributory, go to two referees who not only provide recommendations regarding acceptance, revision, rejection, etc, but also provide their comprehensive and positive comments for the authors. The comments enable many papers to be raised to an acceptable standard and minimize the rejection of good work for the lack of the art of presentation.

The new era under a reputed publisher and the dedicated editorial team looks very bright and will take the journal to new heights. With this, I look forward to your valuable scientific contributions and cooperation.

